# Pericholecystic Fat Stranding as a Predictive Factor of Length of Stays of Patients with Acute Cholecystitis: A Novel Scoring Model

**DOI:** 10.3390/jcm13195734

**Published:** 2024-09-26

**Authors:** Suh-Won Lee, Cheng-Han Tsai, Hui-An Lin, Yu Chen, Sen-Kuang Hou, Sheng-Feng Lin

**Affiliations:** 1 Department of Emergency Medicine, Taipei Medical University Hospital, Taipei 110, Taiwan; 173122@h.tmu.edu.tw (S.-W.L.); b101101122@tmu.edu.tw (C.-H.T.); 133005@h.tmu.edu.tw (H.-A.L.); b101102093@tmu.edu.tw (Y.C.); 2 Department of Emergency Medicine, School of Medicine, College of Medicine, Taipei Medical University, Taipei 110, Taiwan; 3 School of Public Health, College of Public Health, Taipei Medical University, Taipei 110, Taiwan; 4 Department of Public Health, School of Medicine, College of Medicine, Taipei Medical University, Taipei 110, Taiwan; 5 Department of Evidence-Based Medicine, Taipei Medical University Hospital, Taipei 110, Taiwan

**Keywords:** acute cholecystitis, length of stays, Tokyo guidelines, pericholecystic fat stranding, computed tomography

## Abstract

**Background:** The 2018 Tokyo Guidelines (TG18) are used to classify the severity of acute cholecystitis (AC) but insufficient to predict the length of hospital stay (LOS). **Methods:** For patients with AC, clinical factors and computed tomography features, including our proposed grading system of pericholecystic fat stranding were used for predicting an LOS of ≥7 days in the logistic regression models. **Results:** Our multivariable model showed age ≥ 65 years (OR: 2.56, *p* < 0.001), C-reactive protein (CRP) ≥ 2 mg/dL (OR: 1.97, *p* = 0.013), gamma-glutamyltransferase levels (OR: 2.460, *p* = 0.001), TG18 grade (OR: 2.89 per grade, *p* < 0.001), and moderate to severe pericholecystic fat stranding (OR: 2.14, *p* = 0.012) exhibited prolonged LOS ≥ 7 days. **Conclusions:** We developed a scoring model, including TG18 grades (score of 1–3 per grade), our grading system of fat stranding (score of 1), CRP (score of 1), and gamma-glutamyltransferase (score of 1), and a cutoff of >3 had highest diagnostic performance.

## 1. Introduction

Acute cholecystitis (AC) is a common diagnosis for patients presenting in the emergency department with abdominal pain. In general, history taking, physical examination, and abdominal sonography are adequate for making a clinical diagnosis of AC [1]. However, distinguishing between complicated and uncomplicated AC generally requires multislice computed tomography (MSCT). Abdominal MSCT has high sensitivity (85% to 94%) for the detection of AC [2,3]. MSCT plays an essential role in evaluating the severity of AC [4].

The 2018 Tokyo Guidelines (TG18) regarding the diagnostic and severity grading criteria for AC are widely used to classify AC severity. The severity of AC is classified as grade I (mild), grade II (moderate), and grade III (severe) [5]. Patients with signs of cardiovascular, neurological, hematological, respiratory, renal, or hepatic system organ dysfunction are categorized as having grade III (severe) AC. Patients with an elevated white blood cell count (>18,000/mm^3^), a palpable tender mass in the right upper abdominal quadrant, onset > 72 h, or marked local inflammation are categorized as having grade II (moderate) AC. Patients with uncomplicated AC who do not meet the criteria for grades II and III are categorized as having grade I (mild) AC. Patients with grade I or II AC can be treated surgically, whereas those with grade III AC are generally treated using percutaneous transhepatic gallbladder drainage [5,6]. MSCT information is not included in the TG18 diagnostic criteria for AC.

Remarkable MSCT features, such as gallbladder distension, wall thickening, and pericholecystic fat stranding, are valuable for their role in diagnosis [7]. The present study proposes that fat stranding may be associated with AC severity. In addition, other MSCT features may assist in distinguishing between complicated and uncomplicated AC. To our knowledge, few studies have evaluated the use of fat stranding signs to assess AC severity [8].

The present study determined whether fat stranding signs were associated with an increased length of hospital stay (LOS) and developed a simple and accessible scoring system that can be implemented in emergency departments.

## 2. Materials and Methods

### 2.1. Participants and Design

This single-center retrospective study was conducted between 1 January 2018 and 31 December 2020 in the emergency department of Taipei Medical University Hospital, Taipei, Taiwan. This hospital is a tertiary care center. The emergency department of this hospital is run by certified emergency physicians and has approximately 60,000 patient visits per year. Study participants were treated in the usual manner. We used the disease codes of the International Statistical Classification of Diseases, Tenth Revision, Clinical Modification (Supplementary Table S1) to enroll eligible patients. Patients who were aged ≥ 18 years, who presented to our emergency department with a confirmed diagnosis of cholecystitis (including both calculus and acalculous cholecystitis), and who were admitted to a ward for subsequent care were enrolled. Furthermore, the management of AC was by the consensus of the emergency physician and the general surgeon (laparoscopic cholecystectomy, percutaneous transhepatic gallbladder drainage [PTGBD], and endoscopic retrograde cholangiopancreatography [ERCP]). Patients were excluded if their MSCT images were not taken at the emergency department, if they were discharged directly from the emergency department, including if they were discharged against medical advice, or if their final diagnosis was not compatible with cholecystitis. The medical charts of the patients were reviewed by Suh-Won Lee, Cheng-Han Tsai, and Yu Chen. This study was approved by the Joint Institutional Review Board (reference number: N202303059).

### 2.2. Data Collection

Patient information relating to age, sex, vital signs, Glasgow Coma Scale score, and a history of hypertension, diabetes mellitus, chronic kidney disease, liver cirrhosis, or malignant disease was obtained upon the arrival of each patient to the emergency department. Blood samples for laboratory tests, including a complete blood cell count and differential count, C-reactive protein (CRP, normal range < 0.5 mg/dL), total bilirubin, and gamma-glutamyl transpeptidase (normal range 5–40 U/L) levels, were obtained within 2 h of the arrival of each patient to the emergency department. TG18 and fat stranding grades were analyzed by reviewing the patients’ data after their discharge. The LOS was considered to be the total number of days spent in the hospital, including the days of the patient’s emergency department stay. The patients were categorized into groups of LOS < 7 and LOS ≥ 7 days.

### 2.3. MSCT Imaging

Abdominal MSCT images were obtained by using the 128-slice SOMATOM Perspective MSCT scanner (Siemens Healthineers Inc., Forchheim, Germany). Scans were obtained from the top of the liver to the pubic symphysis in the pelvic cavity with a 0.625 mm slice thickness. All patients were administered 95 mL of Optiray 350 (Mallinckrodt Medical Inc., Pointe Claire, QC, Canada) contrast medium intravenously. The initial interpretation of the MSCT images was performed by the on-duty emergency physician. All original MSCT images were reviewed by Suh-Won Lee, Cheng-Han Tsai, and Yu Chen to ensure the accuracy of the data.

### 2.4. Classification of Fat Stranding

**Figure 1:**
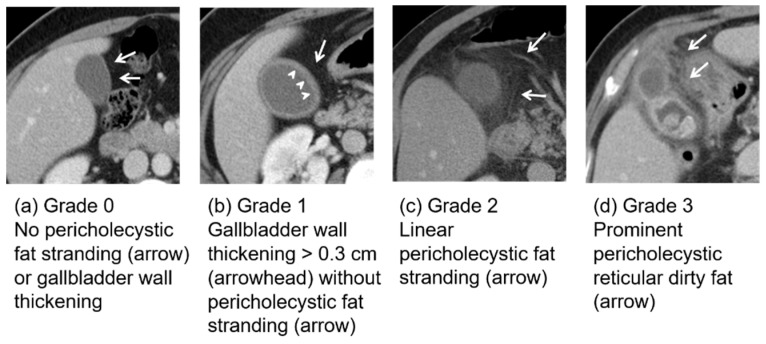
Categorization of fat stranding signs. (**a**) Grade 0: no pericholecystic fat stranding or gallbladder wall thickening. (**b**) Grade 1: gallbladder wall thickening > 0.3 cm without pericholecystic fat stranding. (**c**) Grade 2: linear pericholecystic fat stranding. (**d**) Grade 3: prominent pericholecystic reticular dirty fat.

The gallbladder fat stranding signs were categorized into four grades (Figure 1). Grade 0 was defined as definitely no sign of fat stranding. Grade 1 was considered to be gallbladder wall thickening > 0.3 cm without obvious fat stranding. Grade 2 was considered to be linear fat stranding of the fat adjacent to the gallbladder. Grade 3 was considered to be severe fat stranding extending outside the gallbladder with or without pericholecystic abscess or a gangrene change in the gallbladder wall.

### 2.5. Statistical Analysis

Tests of normality for continuous variables, including the Kolmogorov–Smirnov and the Shapiro–Wilk tests, were performed (Supplementary Table S2). Continuous variables with non-Gaussian distribution were presented as median and interquartile range and were compared using the Mann–Whitney U tests. Categorical variables were presented as proportions and were analyzed using Pearson’s chi-square test or Fisher’s exact test. The cutoff values for age [9,10,11], CRP levels [12], gamma-glutamyltransferase levels, fat stranding, TG18 [13,14], and LOS [15,16,17] were determined with reference to the relevant literature.

Univariable and multivariable logistic regression models were used to obtain the odds ratios (ORs) and corresponding 95% confidence intervals (CIs) for predicting an LOS of ≥7 days. To identify suitable predictors or variables for our multivariable logistic regression models, we used univariable logistic regression analysis and determined which factors were statistically significant (indicated by *p* < 0.05). All significant factors were incorporated into the multivariable logistic regression model. To construct our scoring system, we assigned point values to different potential predictors on the basis of their ORs. The diagnostic performance of each model was obtained by calculating the areas under the curve (AUCs) of the receiver operating characteristics curves. The Youden index was used to determine the optimal cutoff values (the point with the maximum value of sensitivity + specificity − 1) for our developed scoring system. A *p* value < 0.05 was considered statistically significant. All statistical analyses were performed using SPSS version 20 (IBM, Armonk, NY, USA).

## 3. Results

A total of 390 patients with AC were included in this study. There were 324 of 390 (83.1%) patients who had the first episode of cholecystitis; the remaining 66 patients had recurrent cholecystitis. The patients were categorized into groups of LOS ≥ 7 and LOS < 7. The patient characteristics are presented in Table 1. The mean age was significantly higher in the LOS ≥ 7 group (70.0 [IQR, 58.0–80.3] years) than in the LOS < 7 group (54.5 [IQR, 43.3–66.8) years; *p* < 0.001). Gallbladder length was greater in the LOS ≥ 7 group (7.3 [IQR, 5.6–9.0] cm) than in the LOS < 7 group (6.9 [IQR, 5.2–8.1) cm; *p* = 0.0079). Gallbladder width was greater in the LOS ≥ 7 group (4.1 [IQR, 3.5–4.7) cm) than in the LOS < 7 group (3.9 [IQR, 3.2–4.4] cm; *p* = 0.0083). Gallbladder size was greater in the LOS ≥ 7 group (30.4 [IQR, 21.3–41.3] cm^2^) than in the LOS < 7 group (26.0 [IQR, 18.0–33.9] cm^2^, *p* = 0.0020). A total of 138 patients in the LOS < 7 days group (58.5%) underwent laparoscopic cholecystectomy. By contrast, only 34 patients (22.1%) in the LOS ≥ 7 days group underwent laparoscopic cholecystectomy. In total, 1 of 172 patients (0.6%) had conversion from laparoscopic cholecystectomy to open surgery. Percutaneous transhepatic gallbladder drainage was more common in the LOS ≥ 7 days group than in the LOS < 7 days group (44.2% vs. 14.0%, respectively; *p* < 0.0001). Endoscopic retrograde cholangiopancreatography was more common in the LOS ≥ 7 days group than in the LOS < 7 days group (27.3% vs. 12.7%, respectively; *p* = 0.0003). The TG18 grades were higher in the LOS ≥ 7 days group (Grade II, III 75.3%) than in the LOS < 7 days group (Grade II, III 50.4%).

### 3.1. Univariate Analysis

Univariate analysis was used to investigate the factors significantly associated with LOS ≥ 7 days. Age, gallbladder width, size, and volume, common bile duct diameter, fat stranding, TG18 grade, and the laboratory markers of CRP and gamma-glutamyltransferase levels were associated with LOS ≥ 7 days (Table 2).

### 3.2. Multivariable Analysis

Multivariate logistic regression analysis revealed that a high TG18 grade (OR: 2.89 per grade, *p* < 0.001), a fat stranding grade > 2 (OR: 2.14, *p* = 0.012), CRP level ≥ 2 mg/dL (OR: 1.97, *p* = 0.013), gamma-glutamyltransferase level ≥ 40 U/L (OR: 2.460, *p* = 0.001), and age ≥ 65 years (OR: 2.56, *p* < 0.001) were independent predictors of LOS ≥ 7 days (Table 3).

### 3.3. Area under the Receiver Operating Characteristics Curve Analysis

**Figure 2:**
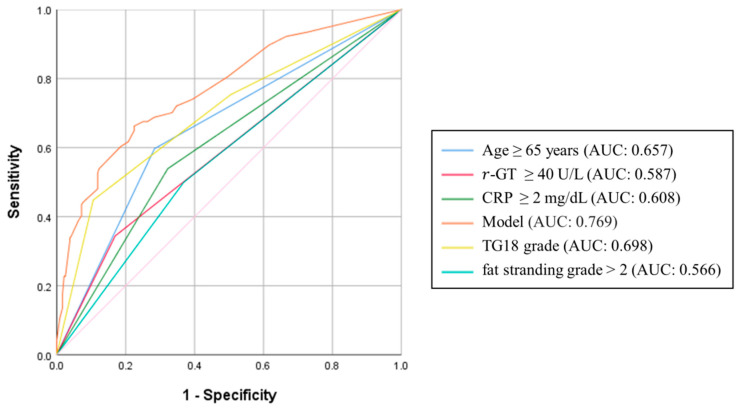
Receiver of operating characteristic curve of the multivariable model.

In a univariate receiver operating characteristics curve analysis (Figure 2), age ≥ 65 years (AUC: 0.657, 95% CI, 0.601–0.713), gamma-glutamyltransferase ≥ 40 U/L (AUC: 0.587, 95% CI, 0.528–0.646), TG18 grade (AUC: 0.698, 95% CI, 0.643–0.752), fat stranding grade > 2 (AUC: 0.566, 95% CI, 0.507–0.624), and CRP level ≥ 2 mg/dL (AUC: 0.608, 95% CI, 0.551–0.666) were comparable for predicting LOS ≥ 7 days. In the multivariable model including the five significant predictors, the diagnostic performance for predicting LOS ≥ 7 days reached a moderate-to-high level (AUC: 0.769, 95% CI, 0.720–0.817). After exclusion of TG18 grade, the multivariable model exhibited a moderate diagnostic performance (AUC: 0.714, 95% CI, 0.662–0.767).

### 3.4. Development of New Scoring System Model

**Figure 3:**
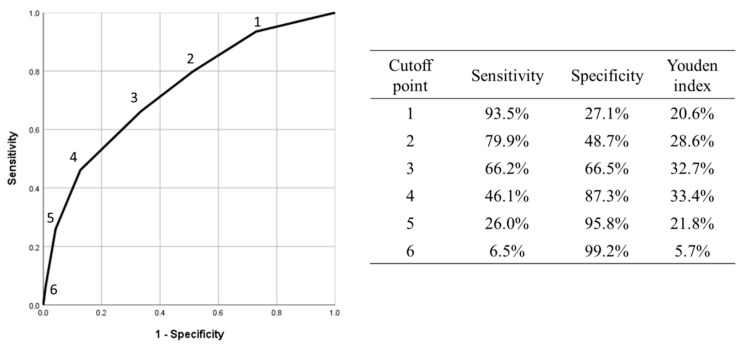
Receiver of operating characteristic curve of the scoring system model.

In our model, TG18 grades I, II, and III were assigned 1, 2, and 3 points, respectively. In addition, fat stranding grade > 2, CRP level ≥ 2 mg/dL, gamma-glutamyltransferase level ≥ 40 U/L, and age ≥ 65 years were assigned 1 point each (Table 3). After the Youden index was applied (Figure 3), a cutoff value of 2 exhibited a sensitivity of 79.9%, a specificity of 48.7%, and a Youden index value of 28.6%. A cutoff value of 3 exhibited a sensitivity of 66.2%, a specificity of 66.5%, and a Youden index value of 32.7%. By contrast, a cutoff value of 4 exhibited a sensitivity of 46.1%, a specificity of 87.3%, and a Youden index value of 33.4%. On the basis of the Youden index results, an optimal cutoff value of >3 was determined.

## 4. Discussion

Our study revealed that older age, elevated inflammatory marker levels, and more pronounced fat stranding signs were associated with a prolonged hospital stay. In our multivariate analysis, age, CRP levels, gamma-glutamyltransferase levels, TG18 grade, and fat stranding were significant predictors for a prolonged hospital stay.

A distinctive feature of our study is the inclusion of fat stranding signs. Our study model’s diagnostic performance was comparable to that of an earlier model that employed ten variables, namely age, sex, body mass index, white blood cell count, neutrophil fraction, platelet count, alanine transaminase levels, admission from the emergency department, gallbladder wall thickening, and pericholecystic fluid levels [18]. In general, severe inflammation of the gallbladder leads to more pronounced fat stranding signs [19,20]. Notably, a novel feature of our study is the proposal of the four grades of fat stranding into a predictive model. Fat stranding is caused by increased congestion and engorgement of lymphatics [21]. Disproportionate fat stranding observed on MSCT is used to evaluate the severity of intra-abdominal inflammation such as acute appendicitis, diverticulitis, and cholecystitis [22]. In case of worsening acute cholecystitis, the gallbladder wall becomes venously engorged and leads to reduced atrial blood flow and subtle ischemic changes. As inflammation progresses, fat stranding becomes more apparent. In addition to fat stranding, our selection of other variables was well justified. The cutoff values we used for LOS, age, CRP levels, and gamma-glutamyltransferase levels were determined based on the relevant literature. First, AC severity is positively correlated with LOS [16], and in most studies, the median LOS was 7 days [15]. Accordingly, LOS ≥ 7 days was defined as a poor outcome (prolonged LOS), while LOS < 7 days was considered a more favorable outcome [16,17]. Second, age was a major risk factor for gallbladder stone formation [23] and increased mortality [24]. Numerous studies have defined age ≥ 65 years as old age [9,10,11]. Third, CRP is considered a strong predictor of complicated AC [13], and a cutoff value of ≥2 mg/dL was reported to exhibit a sensitivity of 71.9% and a specificity of 69.6% [12]. Fourth, gamma-glutamyltransferase levels are elevated in patients with AC, and we selected the upper normal limit value of ≥40 U/L as the cutoff value in our model. Last, TG18 grades are widely used for grading AC severity [14,25]. We assigned scores of 1 to 3 for TG18 grades I to III, respectively, in our model.

In our univariate analysis, the TG18 grades solely had poor-to-moderate diagnostic performance for predicting LOS (AUC: 0.698); however, the multivariable model including age, CRP levels, gamma-glutamyltransferase levels, fat stranding, and the TG18 grades had improved diagnostic performance (AUC: 0.769). To facilitate clinical practice, we constructed a scoring model with a total score of 7. The score was named the FACTT score based on its components: fat stranding, age, CRP levels, TG18, and gamma-glutamyltransferase. Points were allocated based on the OR of each variable. We determined the optimal cutoff value to be a score of 3. Although a score of 4 exhibited the highest Youden index value, it exhibited relatively low sensitivity (46.1%). In contrast, a score of 3 exhibited a sensitivity of 66.2% and a specificity of 66.5%.

Overall, our study’s results are consistent with previous research. Several studies have examined the LOS in patients with AC. For example, patients who received PTGBD before cholecystectomy had a longer LOS [26]. Similarly, another study showed patients receiving PTGBD alone rather than cholecystectomy experienced a longer hospital stay [27]. Our study also found that patients in the LOS > 7 days group were more likely to receive PTGBD (14.0% vs. 44.2%). The duration of symptoms before ED presentation did not significantly affect LOS [28]. Our study similarly found no statistically significant differences in LOS related to the onset time of symptoms before presenting to the ED. The previous literature has shown that increased pericholecystic fat stranding is significantly associated with the severity of AC [29]. In our study, using LOS as a measure of severity for AC, we confirmed that fat stranding is associated with a longer LOS. 

Higher grades of TG18, specifically moderate and severe AC, are associated with an increased complication rate and may necessitate additional imaging studies before surgery for outcome evaluation [5]. MSCT has become the standard imaging modality in most modern EDs. Although sonography is available at the bedside for AC evaluation, it is operator dependent and lacks standardized imaging assessment criteria. In contrast, MSCT benefits from standardized procedures and well-defined imaging protocols, which contribute to more reliable and consistent interpretations of results. This standardization reduces variability and enhances the clarity of diagnostic images, making MSCT a more dependable tool for evaluating the severity of AC. Our model incorporates the novel classification of a pericholecystic fat-stranding sign to address the limitations of TG18.

Our study has several limitations. First, this was a single-center retrospective study. Unmeasured confounding factors remained. Second, patients were of Han Chinese ethnicity; however, we do not believe the clinical course of AC for patients of Han Chinese ethnicity differs to that for patients of other ethnicities.

## 5. Conclusions

Fat stranding, C-reactive protein levels, gamma-glutamyltransferase levels, and age are predictors of length of stays in addition to the 2018 Tokyo Guidelines grade among patients with acute cholecystitis. An increased severity of these factors is associated with more severe acute cholecystitis and longer length of stays. Our simple scoring system is currently being used for the primary evaluation of acute cholecystitis in our emergency department.

## Figures and Tables

**Table 1:** Population characteristics (*N* = 390).

Characteristics	LOS < 7 Days	LOS ≥ 7 Days	*p* Value
Number (*N*)	236 (60.5%)	154 (39.5%)	
Age (years)	54.5 (43.3–66.8)	70.0 (58.0–80.3)	<0.0001 *
Age subgroups			<0.0001 *
20–29 years (*N*)	10/236 (4.2%)	1/154 (0.7%)	
30–39 years (*N*)	28/236 (11.9%)	5/154 (3.3%)	
40–49 years (*N*)	55/236 (23.3%)	15/154 (9.7%)	
50–59 years (*N*)	51/236 (21.6%)	24/154 (15.6%)	
60–69 years (*N*)	43/236 (18.2%)	30/154 (19.5%)	
70–79 years (*N*)	32/236 (13.6%)	40/154 (26.0%)	
≥80 years (*N*)	17/236 (7.2%)	39/154 (25.3%)	
Body mass index (kg/m^2^)	24.6 (22.6–27.3)	24.9 (22.0–27.6)	0.7548
Onset of symptoms to ED (days)	1.0 (1.0–3.0)	1.0 (1.0–3.0)	0.8720
Onset of symptoms to ED > 72 h	30/236 (12.7%)	27/154 (17.5%)	0.1877
ICU admission	8/236 (3.4%)	35/154 (22.7%)	<0.0001
Procedures			
Laparoscopic cholecystectomy	138/236 (58.5%)	34/154 (22.1%)	<0.0001 *
PTGBD	33/236 (14.0%)	68/154 (44.2%)	<0.0001 *
ERCP	30/236 (12.7%)	42/154 (27.3%)	0.0003 *
Imaging findings			
Gallbladder length (cm)	6.9 (5.2–8.1)	7.3 (5.6–9.0)	0.0079 *
Gallbladder width (cm)	3.9 (3.2–4.4)	4.1 (3.5–4.7)	0.0083 *
Gallbladder size (cm^2^)	26.0 (18.0–33.9)	30.4 (21.3–41.3)	0.0020 *
Gallbladder volume (mL)	186.4 (129.9–269.3)	209.1 (135.5–305.0)	0.0863
Gallbladder wall thickness	0.4 (0.3–0.7)	0.5 (0.3–0.7)	0.3118
Calculus cholecystitis	133/236 (56.4%)	82/154 (53.2%)	0.5467
Multiple Gallbladder stone (*N*)	75/236 (31.8%)	52/154 (33.8%)	0.6824
Abscess (*N*)	8/236 (3.4%)	10/154 (6.5%)	0.1533
Grades of pericholecystic fat stranding			0.0518
Grade 0	55/236 (23.3%)	32/154 (20.8%)	
Grade 1	94/236 (39.8%)	45/154 (29.2%)	
Grade 2	44/236 (18.6%)	34/154 (22.1%)	
Grade 3	43/236 (18.2%)	43/154 (27.9%)	
Tokyo guideline grading			<0.0001 *
Grade I	117/236 (49.6%)	38/154 (24.7%)	
Grade II	94/236 (39.8%)	47/154 (30.5%)	
Grade III	25/236 (10.6%)	69/154 (44.8%)	

Continuous variables were expressed as median (interquartile range). Abbreviations: ED, emergency department; ERCP, endoscopic retrograde cholangiopancreatography; PTGBD, percutaneous transhepatic gallbladder drainage. * Statistical significance at *p* value < 0.05.

**Table 2:** Univariate analysis of LOS < 7 days and LOS ≥ 7 days.

Characteristics	Univariate Analysis		
	OR (95% CI)	*p* Value	AUC
Age	1.05 (1.04–1.07)	<0.001 *	0.716
Gallbladder width	1.38 (1.10–1.72)	0.005 *	0.581
Gallbladder size	1.03 (1.01–1.04)	0.001 *	0.596
Gallbladder volume	1.00 (1.00–1.00)	0.034 *	0.555
Gallbladder stone	0.88 (0.59–1.33)	0.546	0.482
Gallbladder wall	1.15 (0.66–2.00)	0.628	0.532
Gallbladder wall HU	1.01 (1.00–1.02)	0.064	0.544
CBD diameter	2.47 (1.26–4.88)	0.009 *	0.589
TG18 grade	2.79 (2.09–3.73)	<0.001 *	0.695
Fat stranding	1.25 (1.03–1.52)	0.022 *	0.563
Fat stranding (0 vs. 1–3)	1.16 (0.71–1.90)	0.558	0.512
Fat stranding (0–1 vs. 2–3)	1.71 (1.13–2.59)	0.010 *	0.563
Fat stranding (0–2 vs. 3)	1.74 (1.07–2.82)	0.025 *	0.549
Abscess	1.98 (0.76–5.13)	0.160	0.516
Gangrene change	1.45 (0.96–2.19)	0.077	0.546
Symptom duration (0–7 and >7)	1.04 (0.92–1.16)	0.561	0.507
Symptom duration (0–7 vs. >7)	0.65 (0.17–2.55)	0.537	0.495
Symptom duration (0–3 vs. >3)	1.46 (0.83–2.57)	0.189	0.524
CRP (normal range <0.5 mg/dL)	1.05 (1.02–1.07)	<0.001 *	0.631
CRP ≥ 0.5 mg/dL	2.26 (1.48–3.46)	<0.001 *	0.598
CRP ≥ 0.8 mg/dL	2.41 (1.59–3.66)	<0.001 *	0.608
CRP ≥ 2 mg/dL	2.46 (1.62–3.74)	<0.001 *	0.608
g-GT (normal range 5–40 U/L)	1.00 (1.00–1.00)	0.007 *	0.556
g-GT ≥ 60 U/L	2.53 (1.53–4.18)	<0.001 *	0.575
g-GT ≥ 40 U/L	2.57 (1.60–4.14)	<0.001 *	0.585

Abbreviations: HU, Hounsfield unit; CBD, common bile duct; CRP, C-reactive protein; g-GT, gamma-glutamyltransferase. * Statistical significance at *p* value < 0.05.

**Table 3:** Multivariate Analysis of LOS < 7 Days and LOS ≥ 7 Days.

Characteristics	OR (95% CI)	*p* Value	Score
TG18 grade (per grade)	2.89 (2.04–4.10)	<0.001 *	1–3 †
Fat stranding grade > 2	2.14 (1.18–3.86)	0.012 *	1
CRP level ≥ 2 mg/dL	1.97 (1.15–3.35)	0.013 *	1
g-GT level ≥ 40 U/L	2.46 (1.44–4.22)	0.001 *	1
Age ≥ 65 years	2.56 (1.59–4.12)	<0.001 *	1

Abbreviations: CRP, C-reactive protein; g-GT, gamma-glutamyltransferase. * Statistical significance at *p* < 0.05. † 1–3 According to TG18, Grade I (mild) = 1, Grade II (moderate) = 2, Grade III (severe) = 3.

## Data Availability

Due to the “Personal Data Protection Act,” the personal health data were not publicly available. Request of the data needs the formal proposal to the Joint Institutional Review Board of Taipei Medical University and the Office of Human Research of Taipei Medical University, Taipei, Taiwan (ohr@tmu.edu.tw).

## Supplementary Materials

The following supporting information can be downloaded at: https://www.mdpi.com/article/10.3390/jcm13195734/s1, Table S1: The List of the International Statistical Classification of Diseases, Tenth Revision, Clinical Modification (ICD-10-CM) of screening all eligible patients aged ≥ 18 years presenting to this ED with confirmed cholecystitis; Table S2. Normality tests of the Kolmogorov-Smirnova and the Shapiro-Wilk’s statistics.
