# Supplementary material for: Pericholecystic Fat Stranding as a Predictive Factor of Length of Stays of Patients with Acute Cholecystitis: A Novel Scoring Model
Source: J Clin Med. 2024 Sep 26;13(19):5734. doi: 10.3390/jcm13195734 (PMC11477346; doi:10.3390/jcm13195734)
Supplement: Supplementary file 1 [file jcm-13-05734-s001.zip › jcm-3204918-supplementary.pdf]

Supplemental Table S1.
The List of *the International Statistical Classification of Diseases, Tenth Revision, Clinical Modification (ICD-10-CM)* of screening all eligible patients aged ≥18 years presenting to this ED with confirmed cholecystitis

ICD-10	ICD-9	Disease names
K81.0	575.0	acute cholecystitis
K81.1	575.11	Chronic cholecystitis
K81.2	575.12	Acute cholecystitis with chronic cholecystitis
K81.9	575.10	Cholecystitis, unspecified
K80.40	574.30/ 574.40	Calculus of bile duct with cholecystitis, unspecified, without obstruction
K80.41	574.31/ 574.41	Calculus of bile duct with cholecystitis, unspecified, with obstruction
K80.42	574.30	Calculus of bile duct with acute cholecystitis without obstruction
K80.43	574.31	Calculus of bile duct with acute cholecystitis with obstruction
K80.44	574.40	Calculus of bile duct with chronic cholecystitis without obstruction
K80.45	574.41	Calculus of bile duct with chronic cholecystitis with obstruction
K80.46	574.30	Calculus of bile duct with acute and chronic cholecystitis without obstructio
K80.47	574.31	Calculus of bile duct with acute and chronic cholecystitis with obstructio
K80		Cholelithiasis
K80.0		Calculus of gallbladder with acute cholecystitis
K80.00	574.00	…… without obstruction
K80.01	574.01	…… with obstruction
K80.1		Calculus of gallbladder with other cholecystitis
K80.10	574.10	Calculus of gallbladder with chronic cholecystitis without obstruction
K80.11	574.11	Calculus of gallbladder with chronic cholecystitis with obstruction
K80.12	574.10	Calculus of gallbladder with acute and chronic cholecystitis without obstruction
K80.13	574.11	Calculus of gallbladder with acute and chronic cholecystitis with obstruction
K80.18	574.10	…… without obstruction
K80.19	574.11	…… with obstruction
K80.2		Calculus of gallbladder without cholecystitis

K80.20	574.20	…… without obstruction
K80.21	574.21	…… with obstruction
K80.6		Calculus of gallbladder and bile duct with cholecystitis
K80.60	574.70	…… unspecified, without obstruction
K80.61	574.71	…… unspecified, with obstruction
K80.62	574.60	Calculus of gallbladder and bile duct with acute cholecystitis without obstruction
K80.63	574.61	Calculus of gallbladder and bile duct with acute cholecystitis with obstruction
K80.64	574.70	Calculus of gallbladder and bile duct with chronic cholecystitis without obstruction
K80.65	574.71	Calculus of gallbladder and bile duct with chronic cholecystitis with obstruction
K80.66	574.80	Calculus of gallbladder and bile duct with acute and chronic cholecystitis without obstruction
K80.67	574.81	Calculus of gallbladder and bile duct with acute and chronic cholecystitis with obstruction
K80.80	574.20	Other cholelithiasis without obstruction
K80.81	574.41	Other cholelithiasis with obstruction
K82.0	575.2	Obstruction of gallbladder
K82.8	575.8	Other specified diseases of gallbladder
K82.9	575.9	Disease of gallbladder, unspecified
K82.A1		Gangrene of gallbladder in cholecystitis
K82.A2		Perforation of gallbladder in cholecystitis

Supplemental Table S2. Normality tests of the Kolmogorov-Smirnova and the Shapiro-Wilk's statistics.

	Kolmogorov-Smirnova		Shapiro-Wilk	
	statistics	*p* value	statistics	*p* value
Age	0.047	0.042*	0.99	0.013*
Body mass index	0.056	0.006*	0.974	<0.001*
Gallbladder length	0.047	0.040*	0.989	0.006*
Gallbladder volume	0.079	<0.001*	0.933	<0.001*
Gallbladder size	0.053	0.010*	0.966	<0.001*
Gallbladder width	0.037	0.200	0.996	0.399
Gallbladder wall thickness	0.158	<0.001*	0.833	<0.001*
Onset of symptoms to ED	0.448	<0.001*	0.075	<0.001*

Abbreviations: ED, emergency department
